# Supplementary figures and images for: A conserved enzymatic toolkit targeting host cell metabolism is associated with Cryptococcus neoformans intracellular survival in protozoal and mammalian phagocytic cells
Source: PLoS Pathog. 2025 Dec 26;21(12):e1013787. doi: 10.1371/journal.ppat.1013787 (PMC12758812; doi:10.1371/journal.ppat.1013787)

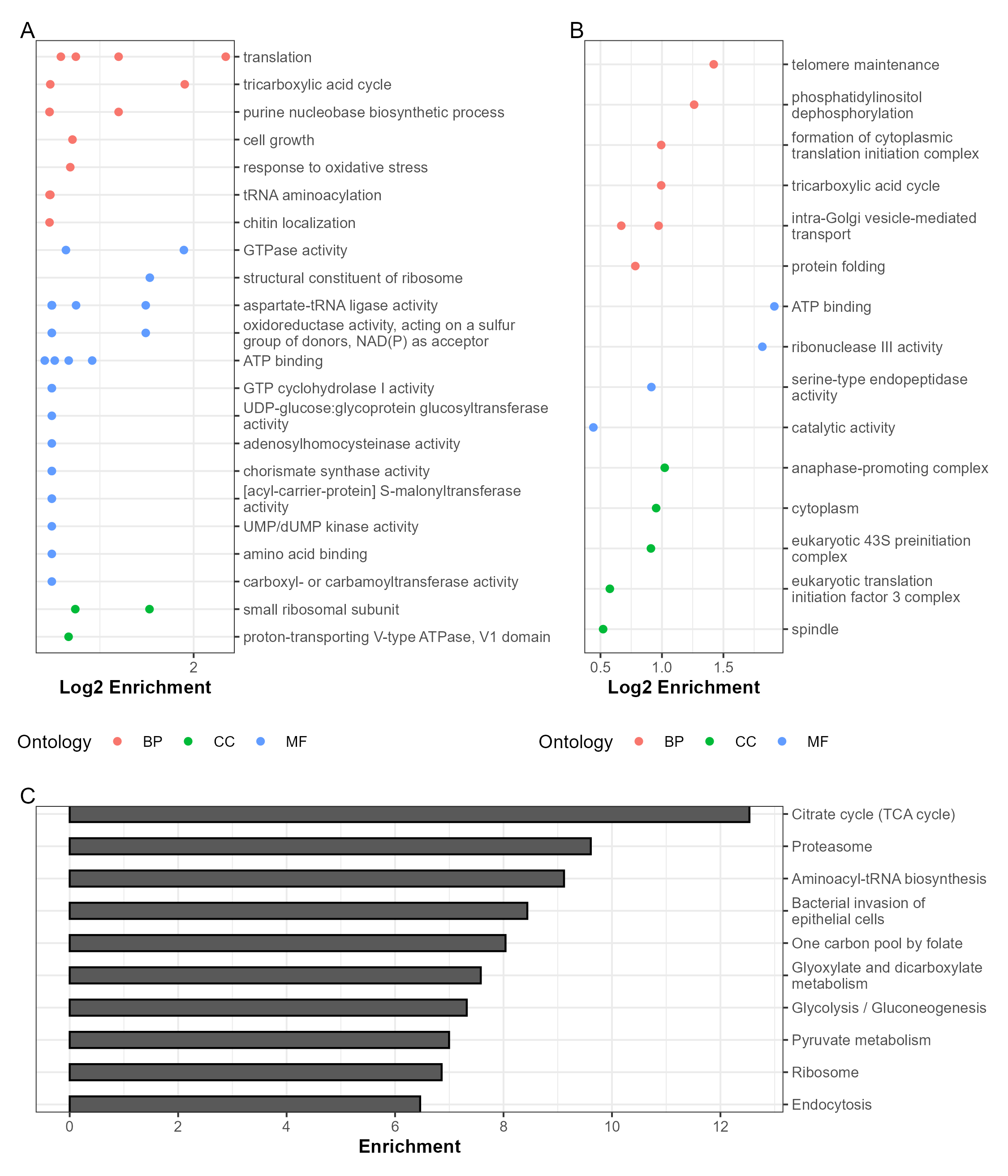

Supplement: S1 Fig — A. GO enrichment analysis of the entire list of 359 peptides identified across all three host species of cell lysates. Enrichment and scoring are based on Fishers’ exact test on the weight01 algorithm. B. GO enrichment analysis of the entire list of proteins identified across polarizations of mouse and human phagocytes. Enrichment and scoring are based on Fishers’ exact test on the weight01 algorithm. Results are consolidated to parental terms. C. KEGG pathway analysis of proteins identified across host species based on Wilcoxon rank sum test. MF: Molecular Function, BP: Biological Process, CC: Cellular Component ontologies. (TIF) [file ppat.1013787.s001.tif]

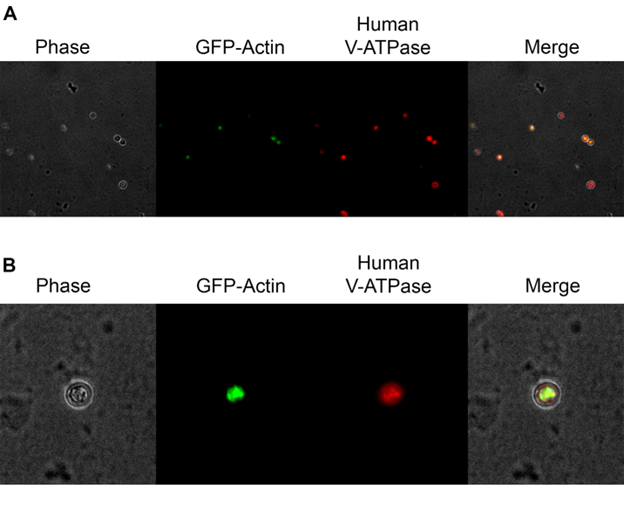

Supplement: S2 Fig — A) 40x isolated phagosomes. Phase contrast, GFP-Actin C. neoformans, stained with 1:100 anti-V-ATPase E1 polyclonal antibody (PA5–29899) and counterstained with Goat Anti-Rabbit IgG H&L conjugated to a Texas Red fluorophore (ab6719) B) 63X isolated phagosome. (TIF) [file ppat.1013787.s002.tif]

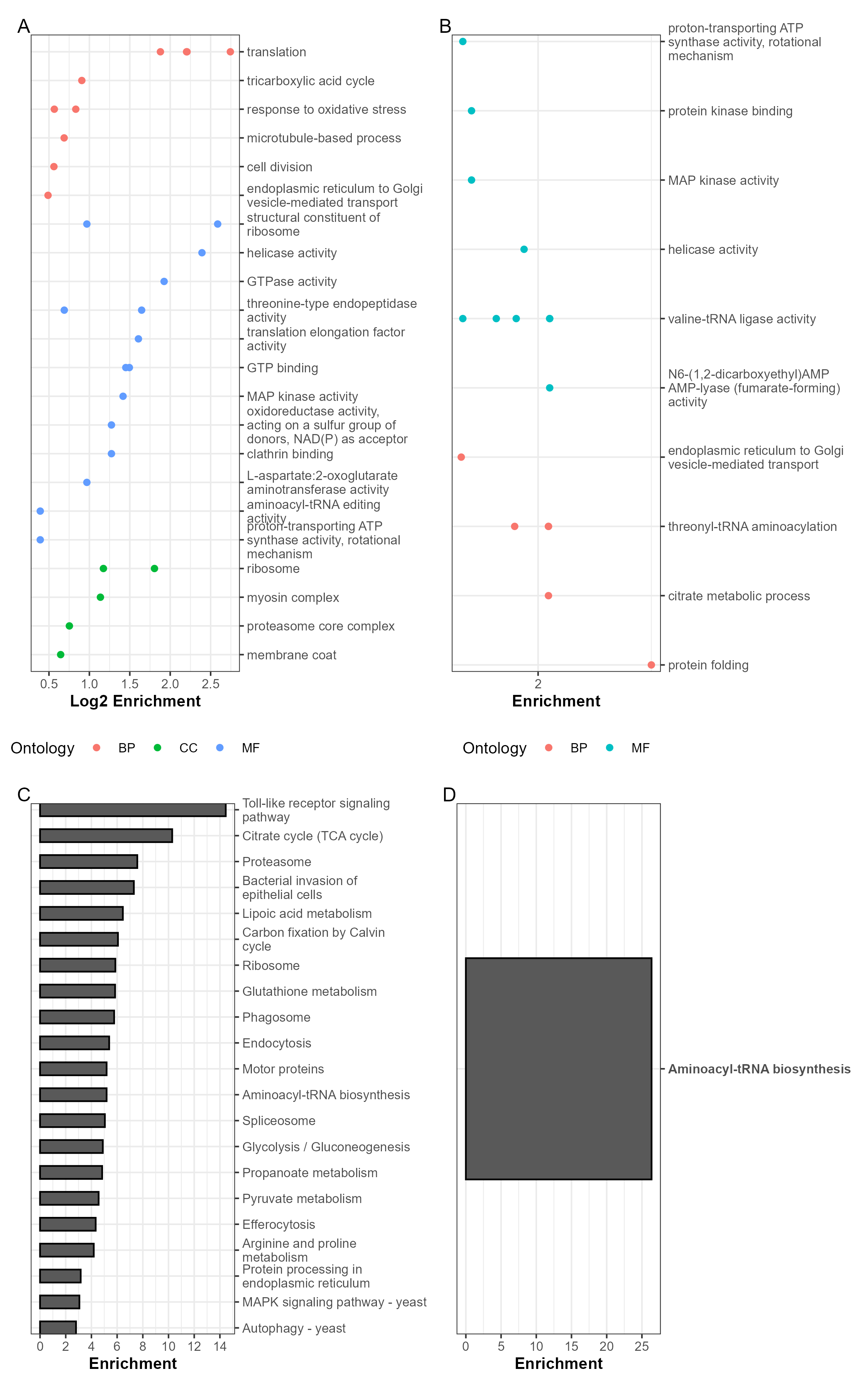

Supplement: S3 Fig — A. GO enrichment analysis of the entire list of 681 proteins identified across all three host species of isolated phagolysosomes. Enrichment and scoring are based on Fishers’ exact test on the weight01 algorithm. B. GO enrichment analysis of the entire list of 42 proteins identified in both phagolysosome and lysate samples. Enrichment and scoring are based on Fishers’ exact test on the weight01 algorithm. Results are consolidated to parental terms. C. KEGG pathway analysis of proteins from isolated phagolysosomes based on Wilcoxon rank sum test. D. KEGG pathway analysis of proteins identified in both phagolysosomes and lysates based on Wilcoxon rank sum test. MF: Molecular Function, BP: Biological Process, CC: Cellular Component ontologies. (TIFF) [file ppat.1013787.s003.tiff]

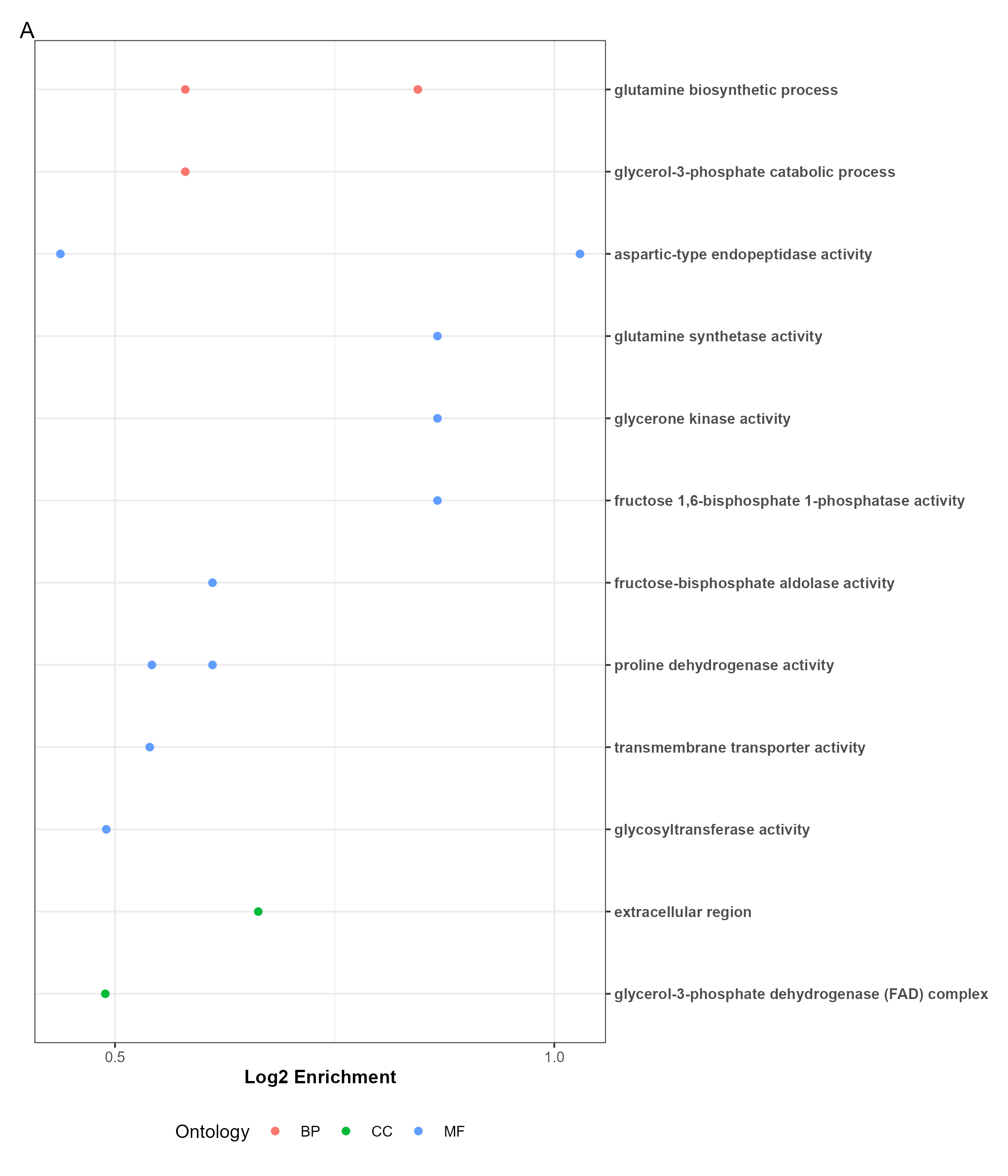

Supplement: S4 Fig — Enrichment and scoring are based on Fishers’ exact test on the weight01 algorithm. Results are consolidated to parental terms. MF: Molecular Function, BP: Biological Process, CC: Cellular Component ontologies. (TIFF) [file ppat.1013787.s004.tiff]

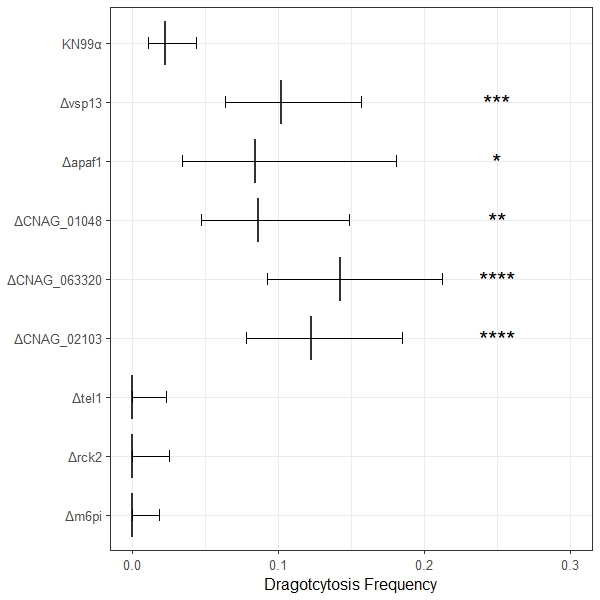

Supplement: S5 Fig — Dragotcytosis frequency is modulated during in vitro BMDM infection of several genes identified as significant in this list but largely absent from wider literature. Error bars represent 95% CI of mean frequency. Significance was determined via test of equal proportions compared to wild-type KN99α and corrected for multiple hypothesis via Benjamini-Hochberg. *, **, ***, and **** denote P < 0.05, 0.01, 0.001, and 0.0001, respectively. (JPEG) [file ppat.1013787.s005.jpeg]

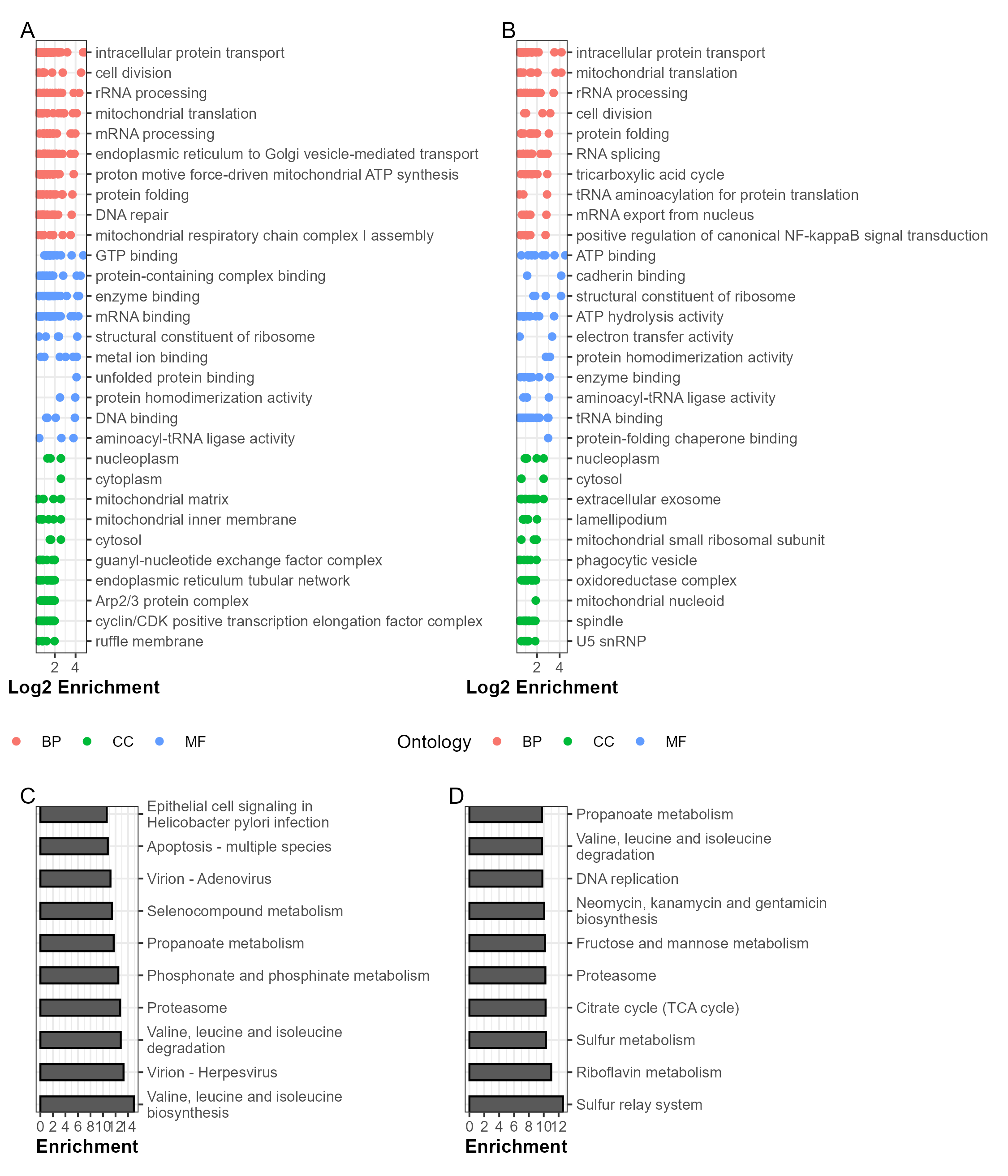

Supplement: S6 Fig — A. GO enrichment analysis of the entire list of 7933 human proteins identified from isolated phagolysosomes. Enrichment and scoring are based on Fishers’ exact test on the weight01 algorithm. B. GO enrichment analysis of the entire list of 8038 mouse proteins identified from isolated phagolysosomes. Enrichment and scoring are based on Fishers’ exact test on the weight01 algorithm. Results are consolidated to parental terms. C. KEGG pathway analysis of human proteins from isolated phagolysosomes based on Wilcoxon rank sum test. D. KEGG pathway analysis of mouse proteins identified in both phagolysosomes and lysates based on Wilcoxon rank sum test. Both GO and KEGG analyses are limited to the 10 most highly represented groups for visualization with complete results in the supplement. MF: Molecular Function, BP: Biological Process, CC: Cellular Component ontologies. (TIFF) [file ppat.1013787.s006.tiff]

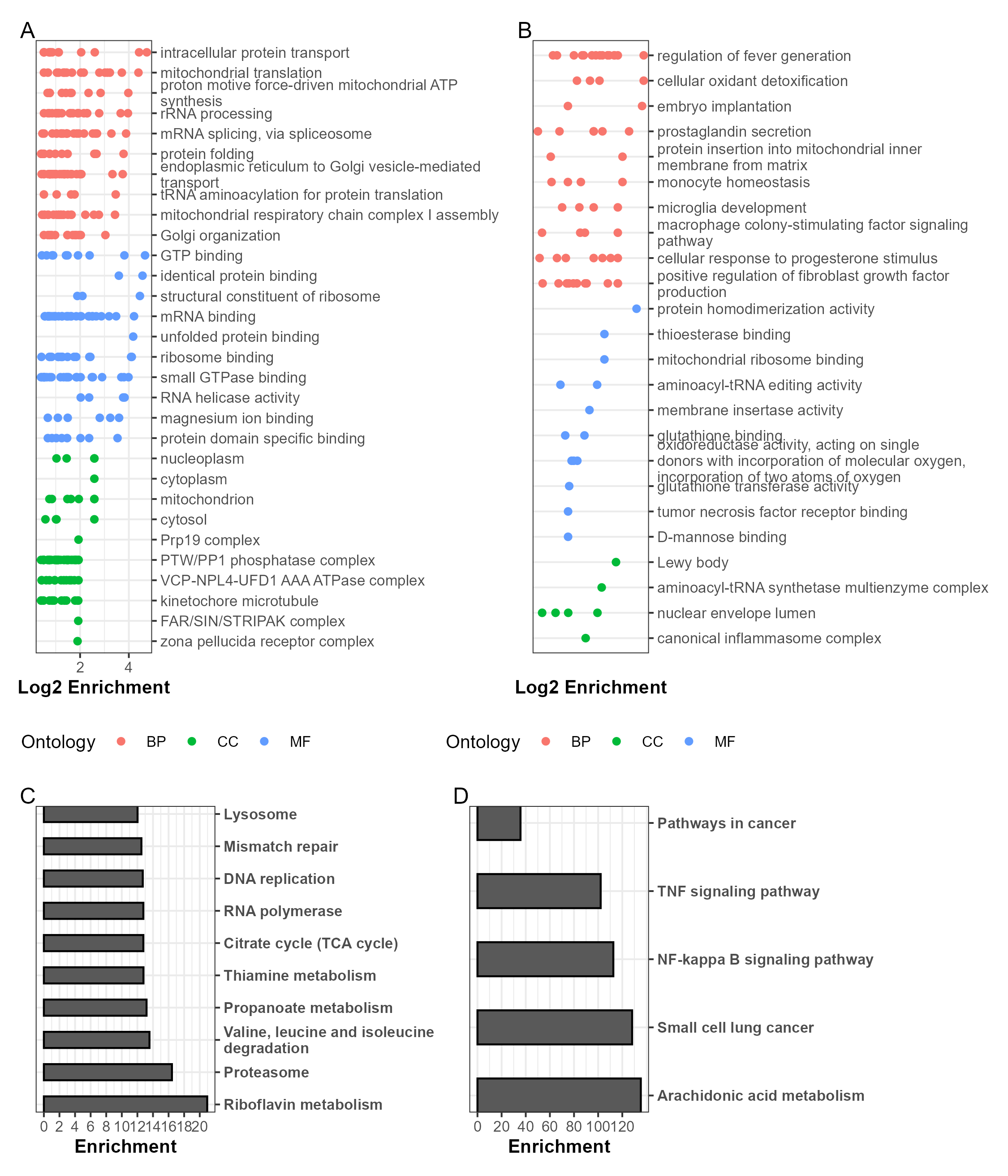

Supplement: S7 Fig — A. GO enrichment analysis of the entire list of 3279 proteins identified from isolated phagolysosomes in both species that are known orthologues. Enrichment and scoring are based on Fishers’ exact test on the weight01 algorithm. B. GO enrichment analysis of the 15 orthologues detected in different abundances between M1 and M2 polarization in both species. Enrichment and scoring are based on Fishers’ exact test on the weight01 algorithm. Results are consolidated to parental terms. C. KEGG pathway analysis of the entire list of known orthologues based on Wilcoxon rank sum test. D. KEGG pathway analysis of the 15 commonly different proteins based on Wilcoxon rank sum test. Both GO and KEGG analyses are limited to the 10 most highly represented groups for visualization with complete results in the supplement. MF: Molecular Function, BP: Biological Process, CC: Cellular Component ontologies. (TIFF) [file ppat.1013787.s007.tiff]
