# Supplementary material for: A conserved enzymatic toolkit targeting host cell metabolism is associated with Cryptococcus neoformans intracellular survival in protozoal and mammalian phagocytic cells
Source: PLoS Pathog. 2025 Dec 26;21(12):e1013787. doi: 10.1371/journal.ppat.1013787 (PMC12758812; doi:10.1371/journal.ppat.1013787)
Supplement: Attachment — Submitted filename: PLOS_Revision_Comments_Responses-02.docx [file ppat.1013787.s035.docx]

PPATHOGENS-D-25-01267
A Conserved Enzymatic Toolkit Targeting Host Cell Metabolism is Associated with Cryptococcus neoformans Intracellular Survival in Protozoal and Mammalian Phagocytic Cells
PLOS Pathogens

Dear Dr. Dragotakes,

Thank you for submitting your manuscript to PLOS Pathogens. The manuscript was reviewed by three experts in the field and although all of them felt it has merit, they all stated that there are several minor issues that need to be addressed before publication. Therefore, we invite you to submit a revised version of the manuscript that addresses the points raised during the review process.

Please submit your revised manuscript within 60 days Sep 27 2025 11:59PM. If you will need more time than this to complete your revisions, please reply to this message or contact the journal office at [plospathogens@plos.org](mailto:plospathogens@plos.org). When you're ready to submit your revision, log on to <https://www.editorialmanager.com/ppathogens/> and select the 'Submissions Needing Revision' folder to locate your manuscript file.

Please include the following items when submitting your revised manuscript:

* A rebuttal letter that responds to each point raised by the editor and reviewer(s). You should upload this letter as a separate file labeled 'Response to Reviewers'. This file does not need to include responses to any formatting updates and technical items listed in the 'Journal Requirements' section below.
* A marked-up copy of your manuscript that highlights changes made to the original version. You should upload this as a separate file labeled 'Revised Manuscript with Track Changes'.
* An unmarked version of your revised paper without tracked changes. You should upload this as a separate file labeled 'Manuscript'.

If you would like to make changes to your financial disclosure, competing interests statement, or data availability statement, please make these updates within the submission form at the time of resubmission. Guidelines for resubmitting your figure files are available below the reviewer comments at the end of this letter.

We look forward to receiving your revised manuscript.

Kind regards,

Felipe H Santiago-Tirado, PhD
Guest Editor
PLOS Pathogens

Michal Olszewski
Section Editor
PLOS Pathogens

Sumita Bhaduri-McIntosh
Editor-in-Chief
PLOS Pathogens
orcid.org/0000-0003-2946-9497

Michael Malim
Editor-in-Chief
PLOS Pathogens
orcid.org/0000-0002-7699-2064

**Additional Editor Comments:**
Dear Dr. Dragotakes, I, as the three reviewers, agree that this is an interesting study that will be of general interest to the cryptococcal field as well to the general microbial pathogenesis community. However, the numerous stylistic concerns (regarding data presentation and description, and confusing methodology) will limit its impact. Moreover, all three of them raised questions about the validity of all the proteins identified: are they really phagosomal proteins? Do they have a physiologically-relevant effect on the host phagosome or the fungus intracellular niche? I believe the limitations of the study need to be clearly stated, especially why "dragotcytosis" was used to validate some of the hits rather than a more relevant assay like phagosomal pH or intracellular replication/survival. Still, we all agree these results will be of broad interest and useful to the field. I look forward to your resubmission.

**Response:** We apologize for some of the confusing methodology. We admit that we found this story difficult to organize and write since it involved comparing three different types of host cells in various conditions. We believe that the proteins identified represent an intracellular cryptococcal secretome. We cannot be sure that these are all phagosomal proteins since the phagosomal membrane is leaky and its contents diffuse into the cytoplasm (PMID: 11880650). We regard to the readout we used Dragotcytosis because it is complex process that includes both the host and the microbe, which is contrast to simpler readouts such as intracellular killing. Phagosomal acidification was not used since we had exhaustively studied it in a prior publication (PMID: 32298242). We appreciate the editor noted that the results would be of broad interest to the field and note that paper contains the first explanation on how fungal cells achieve the capacity for being generalists in being able to infect diverse cell hosts.

**Reviewers' Comments:**

Reviewer's Responses to Questions

**Part I - Summary**
Please use this section to discuss strengths/weaknesses of study, novelty/significance, general execution and scholarship.

Reviewer #1: In this manuscript Dragotakes et al. have amassed a dataset of proteomics and transcriptomics aimed at further understanding the interaction of C. neoformans with phagocytes including macrophages (mouse BMDM), monocytes (human PBMC), and amoeba (Acanthamoeba castellanii). Specifically, the investigators are interested in how the Cryptococcal response to being eaten by these different types of cells is similar or different. The investigators conclude that while there are situation-specific differences, much of the Cryptococcal response to being eaten is shared in the models. Overall, it’s a straightforward premise and an interesting pile of data. I have only modest further questions/concerns:

**Response:** We appreciate that the reviewer found the premise of our study interesting.

Reviewer #2: Here, the authors successfully adapted a methodology for isolation of phagolysosomes that improved the identification of fungal cells after been in contact with host cells. This paper also analyses common routes employed by C. neoformans to defend themselves against predators and immune cells. In general, the paper is well written and organized. However, in order to improve its quality, some minor details should be addressed. For example, several results are described very lightly which forces the reader to go constantly to see the figure in question and halts the readability of this paper. Furthermore, the authors use several specific terminologies and abbreviations that need be explained to help the reader to understand the results. Lastly, there is no description in the figure caption related to statistical analysis and softwares used to create the images that limits the reproducibility of the results.

**Response:** We appreciate the reviewer comments and have strived to provide more details in figures and methodology in the revised manuscript to clarify the results.

Reviewer #3: The manuscript by Dragotakes et al describes a proteomics and transcriptional response to phagocytosis of C. neoformans by mouse and human macrophages and Amoeba. The fascinating discovery is that the process of phagosome formation is highly similar among these species, suggesting that C. neoformans has evolved to trigger similar responses in phagocytes of vastly different origins. The paper is well-laid out and largely focuses on the results of multiple proteomics and transcriptional experiments. Overall, this is a large undertaking where these data will be valuable to the scientific community. My comments are largely minor in nature but should be addressed prior to consideration of publication.

**Response:** We are happy to read that Reviewer 3 found the discovery of commonalities in phagosomal formation across three very different host species ‘fascinating’ and have strived to respond to her/his comments to improve readability. We also appreciate that the reviewer noted that this was a ‘large undertaking’ since it took us several years to both figure out how to do these experiments and accumulate the data presented in this paper.

**Part II – Major Issues: Key Experiments Required for Acceptance**
Please use this section to detail the key new experiments or modifications of existing experiments that should be absolutely required to validate study conclusions.

Generally, there should be no more than 3 such required experiments or major modifications for a "Major Revision" recommendation. If more than 3 experiments are necessary to validate the study conclusions, then you are encouraged to recommend "Reject".

Reviewer #1: N/A

Reviewer #2: Line 145: Since the isolation of C. neoformans containing phagolysosomes is so problematic and the protocol was adapted from aspergillus to C. neoformans. Briefly describe changes for C. neoformans?

**Response:** We have updated the text to be more thorough. Although as described in (36247839) we struggled for a decade to isolate the cryptococcal phagosome. The aspergillus protocol required very few changes to work with *C. neoformans*, so we felt applied is a more apt term to use than adapted.

Line 258: Define Dragocytosis? This term is mentioned several times throughout the manuscript but is only briefly described at the end of the discussion. An earlier definition would improve the readability of the paper.

**Response:** Dragotcytosis is a process of simultaneous non-lytic exocytosis by C. neoformans followed by an ingestion event in another cell resulting in macrophage cell to cell transfer, and was described in (PMID: 30877168). We have updated the text to include an earlier description of Dragotcytosis.

Line 268: Provide more details about number of proteins, examples of pathways, etc? Furthermore, please describe each panel individually.

**Response:** We have expanded the description of these results.

Line 271-273: authors state that there is consistency of cryptotoccal peptides among species but there are clear differences in expression levels between Mm and Hs in Fig 1A and between humans and mouse and amoeba in Fig 1B.

**Response:** We have updated the text to be more consistent. While there are differences in abundances, we were surprised by the overall consistency of identified proteins and the direction of significant gene expression. We expected most identified proteins, and especially DEGs, to be much more varied between host macrophages and polarization states so were surprised to see such an overall consistency. The heatmap in Figure B actually shows normalized values across each row according to relative abundance of identified proteins. Taken at face value it appears there are large differences in Mice and Human samples but we are unable to establish statistical significance for most of those differences. The bar on the left shows were we do see differences between human and mouse samples and they are less stark.

Line 301: The 267 proteins differently abundant are in which condition? There are several comparisons in figure 3. Furthermore, please describe each panel individually.

**Response:** We updated the text to reflect which specific comparisons we were referencing in these parts.

Fig 1-3: Are the authors showing peptides or proteins? Although proteomics measures peptides, normally in bottom-up analysis like this, original proteins are traced back and showed. Likewise, if peptides, elaborate on the concept of expression levels for peptides?

**Response:** The reviewer is correct, for the actual analysis we relied on proteins rather than peptides for the analyses. We have updated text to be clearer about this.

Provide the meaning of abbreviations used, such as MM, Hs and Ac? Same about AR in X axis? Briefly describe which software and statistical analysis were used to generate the figures? Is the abundance relative? If so, please state that.

**Response:** We have updated the figure legend to explain each acronym/abbreviation.

Reviewer #3: 1. The time for phagocytosis is not clear. For the proteomics experiments, it suggests that C. neoformans and phagocytes were exposed for 2 hours. Was there a washout period of non-adhered fungi to ensure that phagosomes were of similar age (as determined in minutes/hours), or was C. neoformans present throughout? If the latter, then phagosomes could be 1 min to 120 minutes old. If the latter, how does one properly interpret the proteomics data.

**Responses:** Thank you for catching this. We synchronize phagocytosis through centrifugation of macrophage containing plates, which force the yeast cells and macrophages to come together in latter monolayer. We had neglected to include this detail in the prior version. We have updated the text to reflect this.

2. For transcriptional analysis, the time of exposure of C. neoformans to phagocytes is not clear.

**Response:** Infections were longer for the transcription analysis, we have updated the text to specify the hours post infection.

3. In all presented data where the authors’ emphasis is that the lists of proteins or upregulated genes are similar, the addition of Venn diagrams to volcano plots/ heat maps would be a great visual aid to make the case (as has been done in prior publications from the Casadevall lab).

**Response:** In experiments where differing proteins/genes are detected we include upset plots rather than Venn diagrams. Since we make up to 18 comparisons at a time, we find the layout more legible. For figures that do not feature an upset plot (Figures 1, 2, 3) the overlap is so thorough we did not include them. We have updated the text in these sections to emphasize this.

**Part III – Minor Issues: Editorial and Data Presentation Modifications**
Please use this section for editorial suggestions as well as relatively minor modifications of existing data that would enhance clarity.

Reviewer #1: In figure 1B can we confirm that the color legend is correct? It looks like mouse and amoeba cluster together, while human is more different, but the rest of the data suggests mouse and human are more similar to each other.

Response: The colors are correct, the clustering here is due to the fact that we are using all the relative abundance of all proteins compared to each other to visualize the data as a whole. Those differences between human and mouse are not statistically significant. If we were to only show the proteins with statistically significant abundance differences between mouse and human they would cluster as the reviewer expects.

It looks like the analyses were typically done with an n = 3 (e.g. figure 1). Can we clarify whether these are technical or biological replicates? Other illustrations of the data can be less clear. Can we clarify in each case the n and whether and how samples were pooled for analysis throughout?

**Response:** Excellent point, yes each experiment was done with 3 biological replicates. We have updated text and figure legends to clarify the specifics.

In the “purified” phagosome analyses of mouse/human proteins, are the investigators worried that the vast majority of these proteins are unlikely to be phagosome-associated? Clearly there are, for example, abundant of mitochondria. What is the evidence that these proteins are even enriched for phagosomal proteins? Or depleted of other organelles. There might be something to be done by comparison to the whole cell lysate data – albeit imperfect?

**Response:** The phagosome fraction would contain both live and dead cryptococcal cells and the presence of the latter could account for the presence of mitochondrial proteins. Unfortunately, we cannot directly compare the whole cell lysate and phagosomal samples as they were run separately. The mass spectrometer we use has a limited number of spaces for samples in any given run and we organized ours to compare polarization states and host species.

Supplementary Figure 1 – define BB, CC, MF: Molecular Function (MF), Biological Process (BP), and Cellular Component (CC).

**Response:** Sorry for the undefined abbreviations. Text updated to define terms.

Table1 and Table2 use differently formatted Gen IDs. Presumably all lower case is preferred.

**Response:** Agreed, lower case is preferable for both tables. The text has been updated.

Line 211: “The horizontal dendrogram shows the proteins in samples that clustered together.” Presumably this was copied from somewhere when a dendrogram was shown.

**Response.** The reviewer is correct. This came about because we changed the visualization while editing (horizontal dendrograms took up too much space) and forgot to update the text.

The text often refers to detecting peptides vs detecting proteins. I’m presuming that the processed data being analyzed throughout relates to peptide data that has already been compiled into quantitative protein data?

**Response:** Yes, this is correct. We have updated the text to clearly differentiate when referring to the isolated peptides vs their respective identified proteins.

The method for polarizing BMDMs and making whole cell lysates is described, but not for the human monocytes? Presumably the same? Any modifications?

**Response:** Correct, the only real difference being the use of human IFN and IL-4, which is now updated in the text.

How were purified phagosomes processed for proteomics? Same lysis approach?

**Response:** Correct and sorry for this omission. The beginning of the proteomics methods section should not have referenced only whole cell lysate and has been updated.

As a final note, I found that the story can be quite confusing to a reader trying to understand specifically when Cryptococcal proteins/genes are being assessed (usually) and when human/mouse/amoeba proteins or genes are being assessed. Any help with more overt and explicit labeling and text might be helpful.

**Response:** We apologize for the lack of clarity. Given three phagocytic hosts and multiple methods there are a large number of potential comparisons in this study and clarity has certainly been our biggest enemy during the preparation of the manuscript. I have tried to update the text more to further clarify when we are talking about which sets of results. In this regard, we found the queries from the reviewers very helpful as they identified areas and procedures that were not clear. We greatly appreciate the reviewers’ input.

Reviewer #2: Line 29-31: citation
Line 68: Elaborate on the explanation of “transfer events”? Added text clarification.
Line 105: pH of acetate buffer? Ac was a poorly chosen acronym on our part for A. castellanii media, we clarified in the text and added pH.
Line 111: complete has an issue with a capital letter. cOmplete is the unfortunately capitalized brand name for the Roche proteinase inhibitor we use.
Line 111: pH of HEPES buffer? Added clarification.
Line 157. Was cell lysis performed without protease inhibitors? In their absence non-tryptic proteases could digest the proteins leading to less protein hits. The lysis method referenced includes the use of cOmplete protease inhibitor.
Line 412: which are “these”? Murine or human’s macrophages? Added clarification, these events are annoyingly rare in both species.

Supplemental Fig 1: Provide the meaning of abbreviations used, such as BB, CC and MF? Added clarification to figure legends.

Why are there different number of points on each row in the enrichment analysis?

Because the terms are consolidated to parental terms each parental term contains varying amounts of children whose enrichment was calculated.

“Ontology is slightly cropped out of the image. Figure has been updated

Caption describes a D panel that does not exist in the image. We have removed the extraneous text, no enriched pathways made our statistical cutoff in this analysis.

Supplemental Fig 3: several labels overlap. Figure has been updated

Line 619: Ref 40: No information about authors, years, journal. Text has been updated

Reviewer #3: Minor point- when human peripheral blood is used, what is the status of IRB approval? Please state if approved or exempt. Exempt statement has been added to the text.

PLOS authors have the option to publish the peer review history of their article ([what does this mean?](https://nam02.safelinks.protection.outlook.com/?url=https%3A%2F%2Ftrack.editorialmanager.com%2FCL0%2Fhttps%3A%252F%252Fjournals.plos.org%252Fplospathogens%252Fs%252Feditorial-and-peer-review-process%2523loc-peer-review-history%2F1%2F010f01985524d69a-f9dcf108-8a3f-4c54-bf8d-50dda42303eb-000000%2FF9fp544NLTH5D4VA0UPsZXrb1ab9_GnSUeRZmgA6zMw%3D221&data=05%7C02%7Cqdragot%40jhu.edu%7C5705235aa58e4eee0f0508ddce73e7c8%7C9fa4f438b1e6473b803f86f8aedf0dec%7C0%7C0%7C638893719420609504%7CUnknown%7CTWFpbGZsb3d8eyJFbXB0eU1hcGkiOnRydWUsIlYiOiIwLjAuMDAwMCIsIlAiOiJXaW4zMiIsIkFOIjoiTWFpbCIsIldUIjoyfQ%3D%3D%7C0%7C%7C%7C&sdata=dN%2B77sHwTiNqSyNK%2FdckQ2crrlQbWP28fiWfmEDdJb4%3D&reserved=0)). If published, this will include your full peer review and any attached files.


If you choose “no”, your identity will remain anonymous but your review may still be made public.


**Do you want your identity to be public for this peer review?** For information about this choice, including consent withdrawal, please see our [Privacy Policy](https://nam02.safelinks.protection.outlook.com/?url=https%3A%2F%2Ftrack.editorialmanager.com%2FCL0%2Fhttps%3A%252F%252Fwww.plos.org%252Fprivacy-policy%2F1%2F010f01985524d69a-f9dcf108-8a3f-4c54-bf8d-50dda42303eb-000000%2F5zd9HCWvlD-A4F3xTCdQbiL92-DWLXiSUxOHxm780Vw%3D221&data=05%7C02%7Cqdragot%40jhu.edu%7C5705235aa58e4eee0f0508ddce73e7c8%7C9fa4f438b1e6473b803f86f8aedf0dec%7C0%7C0%7C638893719420631047%7CUnknown%7CTWFpbGZsb3d8eyJFbXB0eU1hcGkiOnRydWUsIlYiOiIwLjAuMDAwMCIsIlAiOiJXaW4zMiIsIkFOIjoiTWFpbCIsIldUIjoyfQ%3D%3D%7C0%7C%7C%7C&sdata=6mvtX2Qb9dWIkjOls8NZii8DXvZ6kCV8My0%2FZrlOKTk%3D&reserved=0).

Reviewer #1: No

Reviewer #2: No

Reviewer #3: No

[NOTE: If reviewer comments were submitted as an attachment file, they will be attached to this email and accessible via the submission site. Please log into your account, locate the manuscript record, and check for the action link "View Attachments". If this link does not appear, there are no attachment files.]

**Figure resubmission:**

While revising your submission, please upload your figure files to the Preflight Analysis and Conversion Engine (PACE) digital diagnostic tool, <https://pacev2.apexcovantage.com/>. PACE helps ensure that figures meet PLOS requirements. To use PACE, you must first register as a user. Registration is free. Then, login and navigate to the UPLOAD tab, where you will find detailed instructions on how to use the tool. If you encounter any issues or have any questions when using PACE, please email PLOS at [figures@plos.org](mailto:figures@plos.org). Please note that Supporting Information files do not need this step. If there are other versions of figure files still present in your submission file inventory at resubmission, please replace them with the PACE-processed versions.

**Reproducibility:**

To enhance the reproducibility of your results, we recommend that authors of applicable studies deposit laboratory protocols in protocols.io, where a protocol can be assigned its own identifier (DOI) such that it can be cited independently in the future. Additionally, PLOS ONE offers an option to publish peer-reviewed clinical study protocols. Read more information on sharing protocols at <https://plos.org/protocols?utm_medium=editorial-email&utm_source=authorletters&utm_campaign=protocols>
